# Development and validation of a prognostic nomogram for patients with malignant peritoneal mesothelioma

**DOI:** 10.3389/fonc.2025.1480197

**Published:** 2025-02-28

**Authors:** Xiaohan Wang, RuiTing Liu, Chunli Wang, Jingjing Sun, Dongliang Yang

**Affiliations:** ^1^ Chengde Medical University, Chengde, China; ^2^ Department of Ultrasound, Obstetrics and Gynecology Children's Hospital Area, Cangzhou People's Hospital, Cangzhou, China; ^3^ Cangzhou Medical College, Cangzhou, Hebei, China

**Keywords:** malignant peritoneal mesotheliomap, nomogram, overall survival, SEER database, prognosis

## Abstract

**Background:**

Malignant peritoneal mesothelioma(MPM) is a highly aggressive malignant tumor that originates from peritoneal mesothelial cells. Due to the rarity of MPM, there are few survival prediction models specifically for visualization of malignant peritoneal mesothelioma.

**Objective:**

This study aimed to develop a nomogram for the overall survival of MPM based on the Surveillance, Epidemiology, and End Results (SEER) database and the data of Cangzhou People’s Hospital were used for external verification.

**Methods:**

Patients screened from the SEER database were divided into a training group and an internal verification group in a 7:3 ratio, with data from Cangzhou People’s Hospital used as the external verification group. Cox proportional hazard regression was utilized to identify significant factors, and nomograms for 6-month, 12-month, and 18-month overall survival were developed. The performance of the nomogram was assessed using consistency index, calibration curve, and K-M curve.

**Results:**

Age, sex, histology, surgery, tumor size, chemotherapy, differentiated and the number of organ metastases were significant risk factors (p<0.05) and were included in the nomogram.The area under the subject worker curve at 6,12,18 months overall survival (AUC) was 0.782,0.784,0.766 for the training group, 0.804,0.791,0.796 for the internal verification group, 0.767,0.749,0.783 for the external verification group. The predicted correction curve was in good agreement with the observed results. The Kaplan-Meier curves of different risk groups showed significant differences.

**Conclusion:**

This study represents the first visual prognostic model of MPM and the initial incorporation of organ metastasis into MPM prognostic factors. The nomograph serves as a reliable tool for clinicians to personalize overall survival prediction and maximize patient benefits by identifying the most effective treatment.

## 1 Introduction

Malignant peritoneal mesothelioma (MPM) is a highly aggressive tumor derived from the mesothelial cells of the peritoneum. It is clinically uncommon, with an incidence of 0.13/100,000, accounting for 15%-20% of all malignant mesotheliomas (1). In the United States, the incidence is 19.4/106 and 4.1/106 for men and women respectively, with approximately 15,000 new cases diagnosed annually (2). Currently, established carcinogenic factors include chemical agents such as asbestos and other mineral fibers, physical factors like chronic peritonitis and therapeutic radiation, and biological factors. Asbestos is the primary carcinogen in MPM, yet only 33%-50% of patients have a history of asbestos exposure (3). The World Health Organization categorizes this disease into epithelioid, sarcomatoid, and mixed types (4). Most MPM are asymptomatic or asymptomatic in the early stage, with insidious onset. Diagnosis typically occurs during the middle to late stages, and the median time from symptom onset to diagnosis is approximately 4 months. The rarity of peritoneal malignant mesothelioma compared to pleural malignant mesothelioma means that a single institution seldom accumulates a sufficient number of cases for studying the various risk factors. Although some literature (5, 6) has investigated the risk factors for overall survival in malignant peritoneal mesothelioma, the sample size is relatively small and there is currently no visual survival prediction model available for this disease. The typical growth characteristic of PM is extensive growth along the peritoneal surface, but also through local infiltration, implant metastasis, lymphatic and hematogenous metastasis. At present, the prognosis of the distant organ metastasis in patients with malignant peritoneal mesothelioma is still blank. At present, the first-line treatment of MPM is cytoreductive surgery (CRS) combined with hyperthermic intraperitoneal chemotherapy (HIPEC), which can significantly improve the survival of patients. The median overall survival is 19-92 months, and it has become the standard treatment for MPM (7, 8). Most MPM patients are already in advanced stage and have heavy tumor burden, while complete CRS is one of the key factors affecting the survival benefit of patients (9). However, not all patients are suitable for this regimen, and we need individualized treatment based on the condition. Palliative systemic chemotherapy can be used for patients who cannot undergo surgery (10). Due to the rarity of MPeM, there is no specific guideline for systemic chemotherapy of MPeM. Pemetrexed combined with platinum is used as a first-line adjuvant chemotherapy regimen, and other drug combinations are used for second-line treatment (11). Immunotherapy and targeted therapy also show certain potential in malignant peritoneal mesothelioma. How to prolong the survival time of MPM patients and determine the relevant prognostic factors are difficult problems for researchers.

The Surveillance, Epidemiology, and End Results (SEER) database is a population-based cancer reporting system in specific geographic regions of the United States (12). The SEER research data submitted from 17 registries approximately 26.5% of the US population. There were numerous asbestos processing plants in the Cangzhou area of China over 40 years ago, with a significant prevalence of home-style hand-textile asbestos processing, particularly in Heleng City, Dongguang County, Botou City, Xian County and Cangxian County. The Cangzhou People’s Hospital has collected and compiled comprehensive medical records data, which are highly representative sources for obtaining stable and reliable predictive results. Cangzhou People’s Hospital conducted the data collection and construction of the complete medical records. All the above data sources are well representative and can obtain stable and reliable prediction results.

In this study, we aimed to develop and validate a nomogram for predicting the overall survival of patients with malignant mesothelioma using data from the SEER database, and to conduct external validation using patient data from Cangzhou People’s Hospital.

## 2 Methods

### 2.1 Study participants

We used SEER * stat 8. version 4.3 to extract information from the Incidence-SEER Research Data 17 Registries, Nov2022 Sub (2000-2020) database. The Incidence-SEER Research Data 17 Registries contains patient information spanning from 2000 to 2020, encompassing the San Francisco-Oakland SMSA, Connecticut, Hawaii, Iowa, New Mexico, Seattle (Puget Sound), Utah, Atlanta (Metropolitan), San Jose-Monterey, Los Angeles, Alaska Natives, Rural Georgia, and California excluding SF/SJM/LA, Kentucky, Louisiana, New Jersey and Greater Georgia. Patients who meet the inclusion exclusion criteria will be randomized to the training group and the internal validation group in a 7:3 ratio. The 7: 3 ratio has been proved to be a more balanced division method in many practices, which can make the model more robus (13–15) Inclusion criteria: ① Fibrous sarcomatoid (histologic code 9051), Epithelioid (9052), Biphasic (9053), or otherwise unspecified malignant mesothelioma by ICD-O-3 (9054) (16). ② The primary site in the peritoneal, retroperitoneal and retroperitoneal and retroperitoneal overlapping lesions (C48.0-C48.2, C48.8); Only autopsy or death certificate confirmation, no clear diagnosis or incomplete clinical information and survival data were excluded. Similarly, the corresponding patient information obtained from Cangzhou People’s Hospital was included in the external validation team. All participating clinical centers of the SEER platform have received approval from the Institutional Ethics Review Committee. This study has been approved by the Ethics Committee of Cangzhou People’s Hospital. This research align with the principles in the Declaration of Helsinki. Informed consent has been obtained from all participants and/or their legal guardians. Due to the sensitivity of SEER data, in the study, the patient ‘s privacy secrets are strictly protected, such as data collection, storage, analysis and dissemination. The patient ‘s identifiable information is anonymized, and the confidentiality principle is strictly followed in data sharing.

### 2.2 Clinical variables

The present study included the following variables: age; Gender (male and female); Race (white, black, other); Marital status (married, divorced, other), pathology [Sarcomatoid (9051), Epithelial (9052), Biphasic (9053),NOS/other]; Differentiated(Well differentiated, Moderately differentiated, Poorly differentiated, Undifferentiated, Unknown); The primary tumor site (Peritoneum, Retroperitoneum, Overlapping lesion of retroperitoneum & peritoneum); Laterality (Bilateral, Unilateral); Tumor Size; Radiotherapy (Yes,No/Unknown); Chemotherapy (Yes,No/Unknown), Surgery (Palliative, Radical, None, NOS); T-stage (T1, T2, T3, T4, TX); N-stage (N0, N1, NX); M-stage (M0, M1, MX) (17); Bone.metastasis (Yes, No, Unknown); Liver.metastasis(Yes,No,Unknown); Lung.metastasis: (Yes,No,unknown);Number.of.organ.metastases (zero,one,two).The primary outcome was 6-month, 12-month, and 18-month survival in MM patients. OS is defined as the time from diagnosis to death.

### 2.3 Data preprocessing

**Figure 1:**
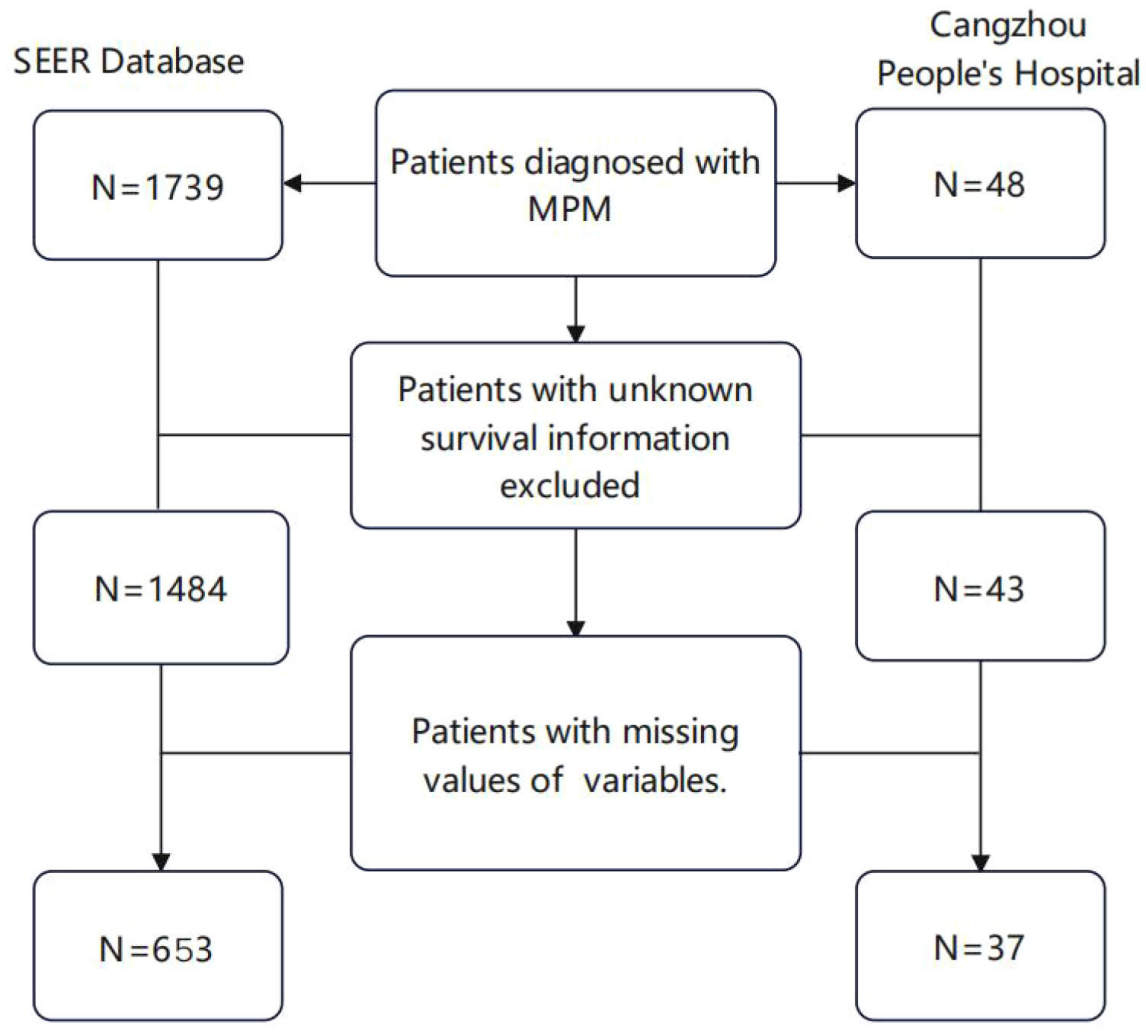
Flow chart of patient screening in the SEER database and Cangzhou People’s Hospital.

Data preprocessing was conducted on a total of 1739 samples, with some cases being excluded based on the predefined exclusion criteria. The process for case selection is illustrated in (**Figure 1**). Age and Tumor Size were input into X-tile software version 3.6.1 to determine the optimal truncation values and convert them into ordered categorical variables. The result displays that the optimal critical value for age is 67 years old (**Supplementary Figure S1**) and the optimal cut-off value for Tumor Size is 80mm (**Supplementary Figure S2**).

### 2.4 Statistical analyses

Statistical analysis was performed using R version 4.4.0. A chi-square test was used to compare the proportion of each categorical variable in the three cohorts. Univariate and multivariate Cox proportional hazards regression analysis was performed in the training cohort. The hazard ratio (HR) and 95% confidence interval (CI) were calculated to evaluate each variable. Variables with statistically significant (p <0.05) were included in the multivariate analysis. A 6-month, 12-month, and 18-month nomogram was utilized to illustrate the impact of statistically significant variables (p<0.05). Further detailed analyses were conducted to assess the performance of the nomogram. The receiver operating curve (ROC) was employed to evaluate the accuracy of nomogram predictions (18).The calibration curves were used to assess the consistency of predicted survival and actual survival. The SEER database and Cangzhou People’s Hospital data were used for internal and external validation, respectively (19).X-tile software was employed to determine the optimal cut-off point based on total patient scores in the training cohort, resulting in stratification of patients into low, medium, and high-risk groups. Based on the total integral of each patient in the training cohort, the optimal cut-off point was determined by the X-tile software to classify the patients in the three cohorts into low, intermediate, and high-risk groups (20). The Kaplan-Meier (K-M) curve was used to describe the overall survival between the different risk groups in the three cohorts.

## 3 Result

### 3.1 The demographic and clinicopathological characteristics of the patients

According to the inclusion and exclusion criteria, a total of 1,739 patients diagnosed with malignant peritoneal mesothelioma were selected from the SEER database. After excluding patients with unknown clinical variables, 648 patients were included in the data analysis and randomly divided into the training group (n=453) and internal validation group (n=195).37 patients from the Cangzhou People’s Hospital were included as the external validation cohort (**Figure 1**).The external validation group exhibited a higher proportion of yellow race (100%), married patients (100%), and non-operative patients (70.3%) compared to the internal validation group and the training group (P<0.001). Distribution of age, sex, histology, laterality, tumor size, primary site, differentiated, tnm stage, chemotherapy, radiation, bone metastasis, liver metastasis, lung metastasis and number of organ metastases had no significant difference among the three cohorts (p>0.05). Detailed information is shown in **Table 1**.

**Table 1:** Clinicopathological characteristics of malignant peritoneal mesothelioma patients in training and validation cohorts.

	External validation	Internal validation cohort	Training cohort	p.overall
	N=37	N=195	N=453	
Age:				0.694
≥67	7 (18.9%)	38 (19.5%)	76 (16.8%)	
18-67	30 (81.1%)	157 (80.5%)	377 (83.2%)	
Sex:				0.26
Female	22 (59.5%)	90 (46.2%)	206 (45.5%)	
Male	15 (40.5%)	105 (53.8%)	247 (54.5%)	
Race:				<0.001
Black	0 (0.00%)	19 (9.74%)	25 (5.52%)	
Other	37 (100%)	9 (4.62%)	30 (6.62%)	
White	0 (0.00%)	167 (85.6%)	398 (87.9%)	
Histology:				0.882
Biphasic	1 (2.70%)	8 (4.10%)	18 (3.97%)	
Epithelial	13 (35.1%)	78 (40.0%)		
Fibrous	3 (8.11%)	9 (4.62%)	19 (4.19%)	
NOS	20 (54.1%)	100 (51.3%)	223	
Tumor.Size:				0.512
<80	1 (2.70%)	14 (7.18%)	23 (5.08%)	
≥80	36 (97.3%)	181 (92.8%)	430 (94.9%)	
Surgery:				<0.001
None	26 (70.3%	115 (59.0%)	283 (62.5%)	
NOS	0 (0.00%)	0 (0.00%)	2 (0.44%)	
Palliative	10 (27.0%)	62 (31.8%)	133 (29.4%)	
Radical	1 (2.70%)	18 (9.23%)	35 (7.73%)	
Laterality:				0.404
Bilateral	1 (2.70%)	7 (3.59%)	9 (1.99%)	
Unilateral	36 (97.3%)	188 (96.4%)	444 (98.0%)	
Primary.Site:				0.915
Overlapping lesion of retroperitoneum & peritoneum	0 (0.00%)	1 (0.51%)	2 (0.44%)	
Peritoneum	36 (97.3%)	188 (96.4%)	440 (97.1%)	
Retroperitoneum	1 (2.70%)	6 (3.08%)	11 (2.43%)	
Marital.status:				<0.001
Divorced	0 (0.00%)	20 (10.3%)	41 (9.05%)	
Married	37 (100%)	118 (60.5%)	260 (57.4%)	
Other	0 (0.00%)	57 (29.2%)	152 (33.6%)	
Radiation:				0.417
No/Unknown	36 (97.3%)	194 (99.5%)	449 (99.1%)	
Yes	1 (2.70%)	1 (0.51%)	4 (0.88%)	
Chemotherapy:				0.827
No/Unknown	13 (35.1%)	78 (40.0%)	173 (38.2%)	
Yes	24 (64.9%)	117 (60.0%)	280 (61.8%)	
Differentiated:				0.521
Moderately	0 (0.00%)	4 (2.05%)	4 (0.88%)	
Poorly	2 (5.41%)	8 (4.10%)	26 (5.74%)	
Undifferentiated	1 (2.70%)	5 (2.56%)	7 (1.55%)	
Unknown	30 (81.1%)	164 (84.1%)	392 (86.5%)	
Well	4 (10.8%)	14 (7.18%)	24 (5.30%)	
T.stage:				0.101
T1	0 (0.00%)	0 (0.00%)	1 (0.22%)	
T2	4 (10.8%)	4 (2.05%)	12 (2.65%)	
T3	5 (13.5%)	15 (7.69%)	38 (8.39%)	
TX	28 (75.7%)	176 (90.3%)	402 (88.7%)	
N.stage:				0.094
N0	11 (29.7%)	23 (11.8%)	68 (15.0%)	
N1	0 (0.00%)	5 (2.56%)	8 (1.77%)	
NX	26 (70.3%)	167 (85.6%)	377	
M.stage:				0.172
M0	5 (13.5%)	13 (6.67%)	37 (8.17%)	
M1	8 (21.6%)	22 (11.3%)	51 (11.3%)	
MX	24 (64.9%)	160 (82.1%)	365	
Bone.metastasis:				1.000
No	37 (100%)	193 (99.0%)	449 (99.1%)	
Yes	0 (0.00%)	2 (1.03%)	4 (0.88%)	
Liver.metastasis:				0.878
No	33 (89.2%)	175 (89.7%)	410 (90.5%)	
Yes	4 (10.8%)	20 (10.3%)	43 (9.49%)	
Lung.metastasis:				0.058
No	37 (100%)	180 (92.3%)	435 (96.0%)	
Yes	0 (0.00%)	15 (7.69%)	18 (3.97%)	
Number.of.organ.metastases:				0.096
one	4 (10.8%)	41 (21.0%)	58 (12.8%)	
two	0 (0.00%)	2 (1.03%)	6 (1.32%)	
zero	33 (89.2%)	152 (77.9%)	389 (85.9%)	

### 3.2 Refinement of variable selection and construction of a nomogram

**Figure 2:**
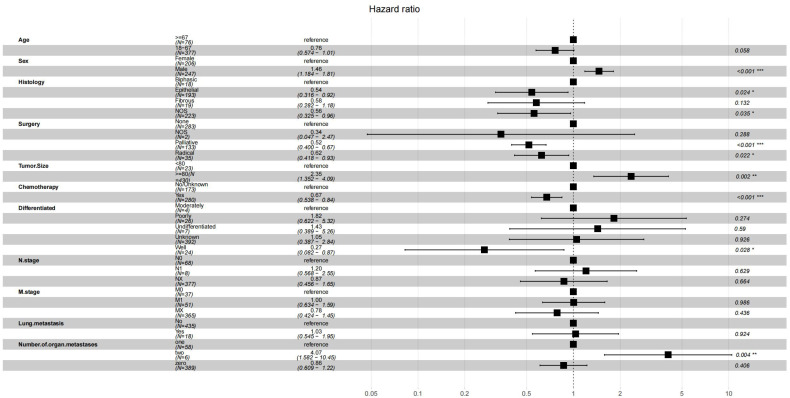
Multivariate analysis of overall survival (OS) in the OS training set.

**Figure 3:**
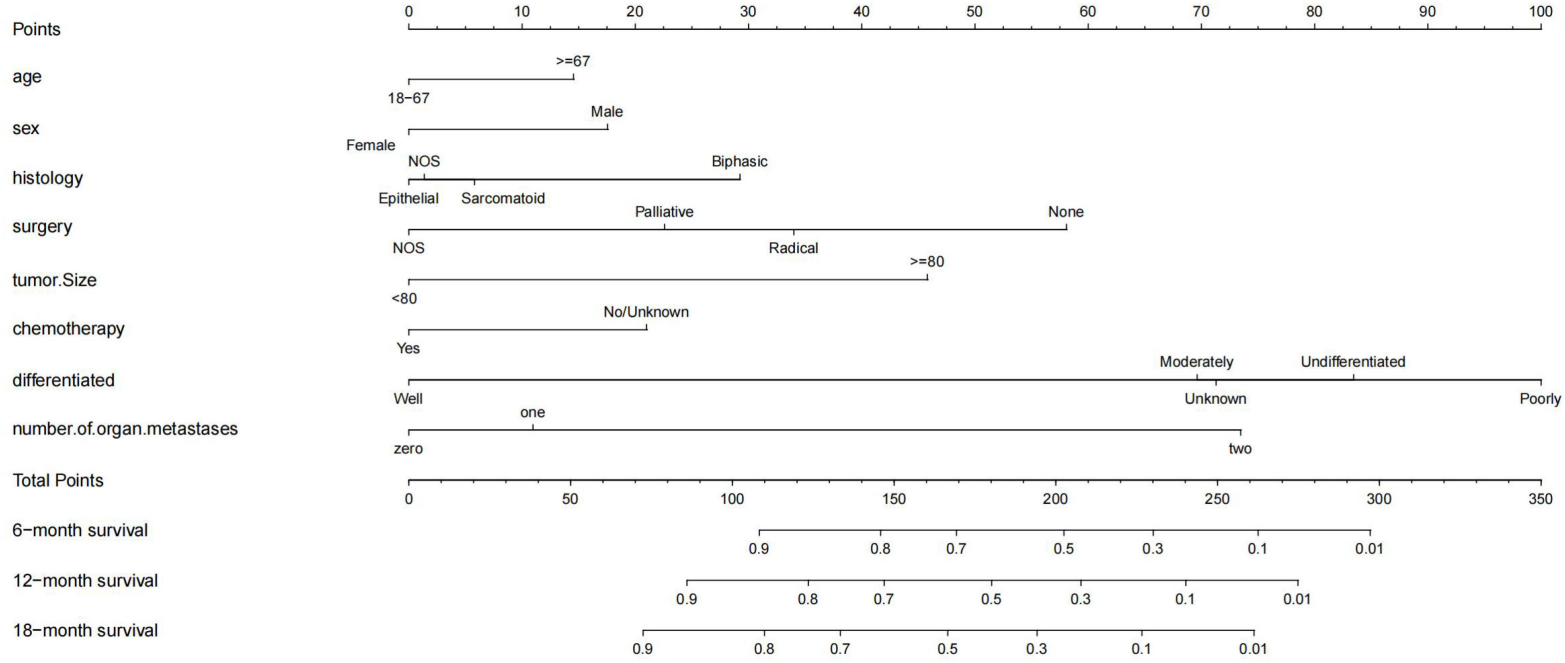
Nomogram for predicting 6-month, 12-month and 18-month overall survival of MPM.

In the Cox regression analysis of single factors, significant correlations were found between age, sex, histology, surgery, tumor size, chemotherapy, differentiation, n stage, m stage, lung metastasis, number of organ metastases and overall survival in malignant peritoneal mesothelioma (p<0.05). Multivariable Cox regression analysis further confirmed that the independent prognostic factors for age, sex, histology, surgery, tumor size, chemotherapy, differentiation, number of organ metastases(p<0.05) (**Figure 2**).The results of univariate and multivariate Cox regression analysis are shown in **Supplementary Table S1**. Based on these prognostic factors, nomograms were developed to assess the overall survival at 6-month, 12-month, and 18-month in MPM patients (**Figure 3**). Each patient was assigned a score for each variable ranging from 0 to 100. The scores for all variables are summed to obtain an overall score, which can be projected onto the bottom coordinate axis of the nomogram.

### 3.3 Evaluation of the nomogram’s performance

**Figure 4:**
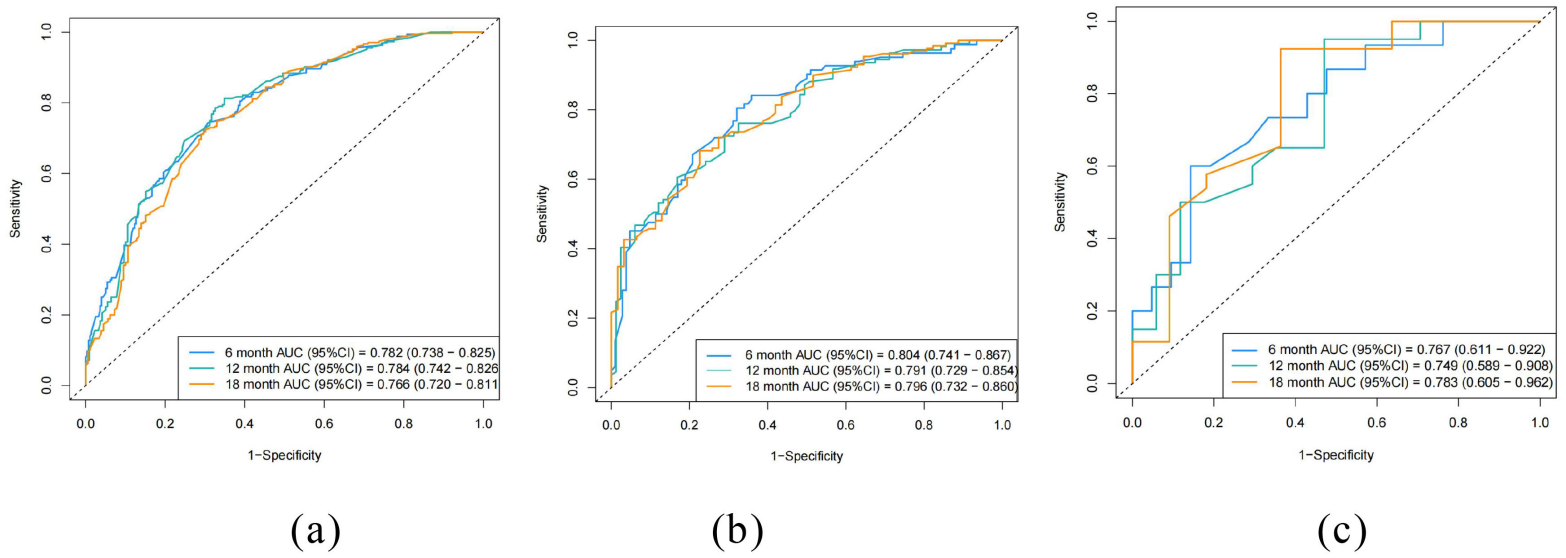
Receiver operating curves (ROC) of the nomogram. **(a)** training cohort, **(b)** internal validation cohort, **(c)** external validation cohort.

**Figure 5:**
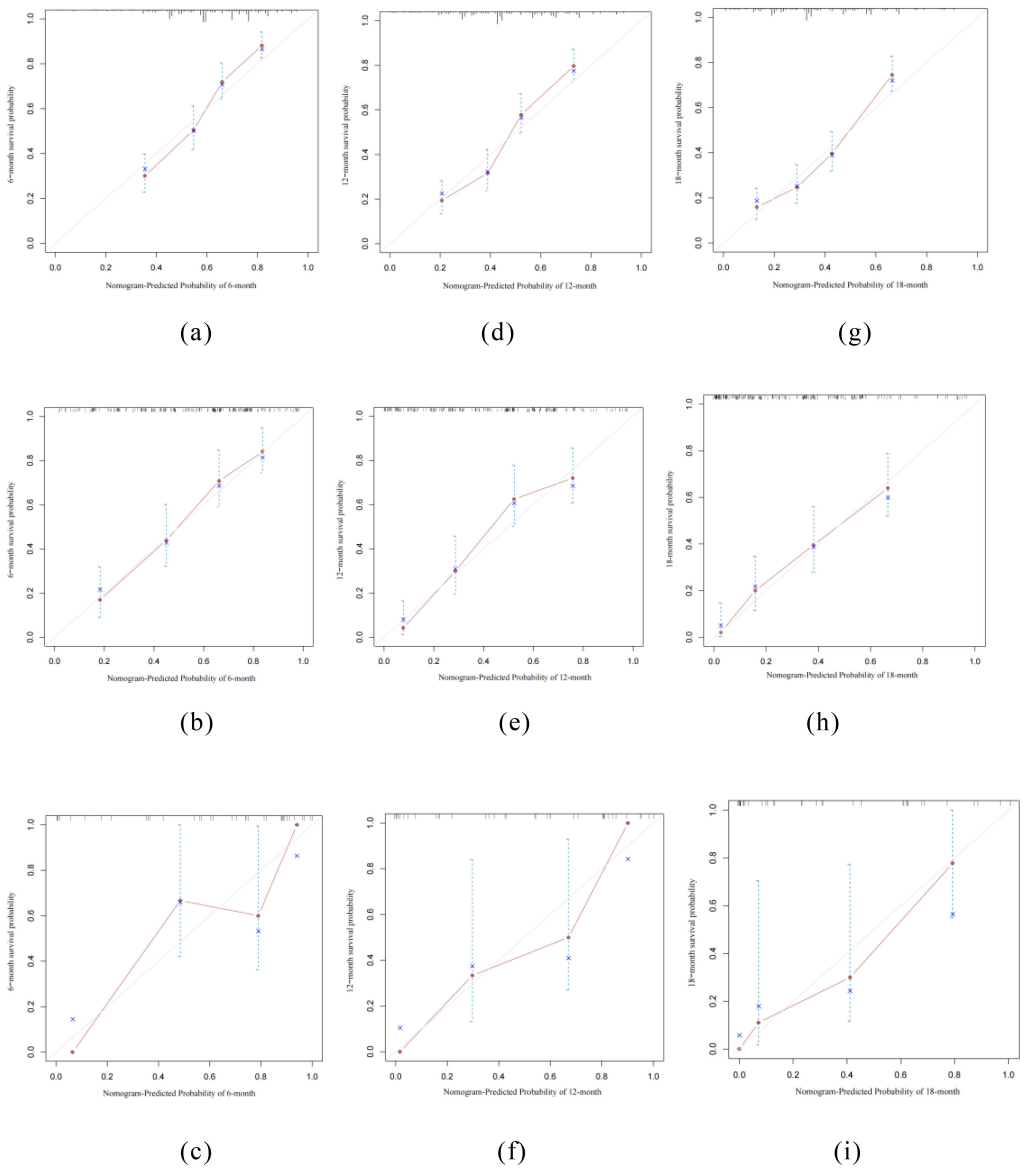
Calibration curves of the nomogram. **(a, d, g)** training cohort, **(b, e, h)** internal validation cohort, **(c, f, i)** external validation cohort.

The ROC curves for the prediction model were plotted at 6, 12, and 18 months (**Figure 4**). The AUC values for the three cohorts in the training group were 0.782, 0.784, and 0.766; in the internal validation group were 0.804, 0.791, and 0.796; and in the external verification group were 0.767, 0.749 and 0.783, indicating strong discrimination ability of the nomogram model. Additionally, calibration curves (**Figure 5**) demonstrated good agreement between the prediction model and reality across all three cohorts.

**Figure 6:**
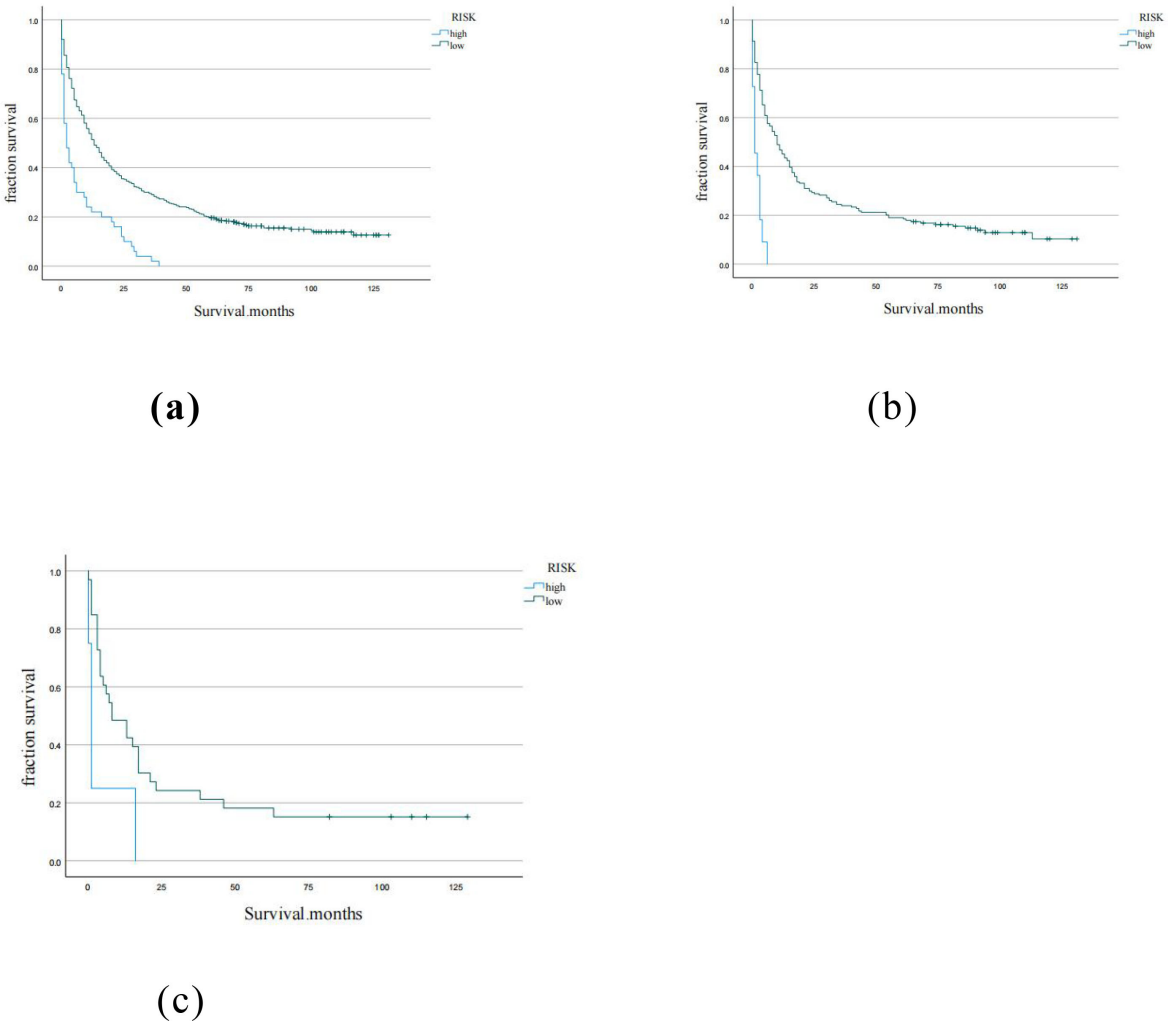
Kaplan-Meier curves for low, moderate, high risk groups in **(a)** training cohort, **(b)** internal validation cohort, **(c)** external validation cohort.

Using the X-tile software, the optimal cutoff for the total score for each patient in the training cohort was 1.4 (**Supplementary Figure S3**). Therefore, patients in each cohort were divided into low risk (total score <1.4) and high risk (total score> 1.4). The K-M curve of OS showed good discrimination between the risk groups in each of the three cohorts (**Figure 6**).

## 4 Discussion

Malignant mesothelioma originates from mesothelial cells retaining multidirectional differentiation potential, often involving the pleural, peritoneal, pericardium and testicular sheath, with the pleura being the most commonly affected site, followed by the peritoneum, accounting for only 7% to 20%. The clinical symptoms of MPeM are not specific and usually include abdominal pain, abdominal distension, weight loss, ascites, and anorexia, and a few symptoms may present with unexplained fever, a hypercoagulable state of the microcirculation, and intestinal obstruction (21). The majority of MPeM patients are advanced at diagnosis and insensitive to chemoradiation, resulting in a poor prognosis and fatal rapid course with a median survival of 6 to 12 months (22)and a mean symptom to survival time of 345 d (23).The first-line treatment for MPM is cytoreduction (cytoreductive surgery, CRS) combined with peritoneal thermoperfusion chemotherapy (hyperthermic intraperitoneal chemotherapy, HIPEC). However, not all patients are suitable for this regimen requiring individualized treatment according on the condition (7).Systemic chemotherapy is an alternative therapy for patients who are not suitable for or wish to undergo non-surgical treatment. Based on the results of a multicenter phase III clinical study involving 456 patients with pleural mesothelioma, the combination of cisplatin and pemetrexed was approved by the Food and Drug Administration (FDA) as the first standard chemotherapy regimen for mesothelioma (24). For MPeM patients who cannot tolerate platinum drugs, pemetrexed + gemcitabine combination therapy may benefit (25). Studies have shown that compared with systemic chemotherapy (cisplatin and pemetrexed), patients treated with the combination of nivolumab and ipilimumab have better clinical benefits (26).In addition, MPM can induce systemic metastasis, with a rate of extraperitoneal metastasis of about 50%. However, due to the rarity of MPeM, there are few visual survival prediction models specifically for malignant peritoneal mesothelioma, and few studies emphasize the role of distant metastasis in the prognosis assessment of malignant peritoneal mesothelioma. This study can make up for the deficiency of related studies and broaden its applicability in different ethnic groups.

In this study, we included patients diagnosed with malignant peritoneal mesothelioma from 2000 to 2020 in the SEER database and from 2018 to 2024 at Cangzhou People’s Hospital. Statistical analysis revealed that age, sex, histology, surgery, tumor size, chemotherapy, differentiation, number of organ metastases were identified as independent prognostic factors for overall survival(p<0.05).Our study findings are in line with prior research, indicating that older age (27), lower the degree of differentiation (28), bigger tumo (29)mean a poorer prognosis; moreover, female prognosis is better than men (27), which may be related to less asbestos exposure, histological type and estrogen receptor expression; Patients with epithelial histological type exhibited a higher survival rate (30), while those with sarcomatoid tissue showed a lower survival rate (31).This study also demonstrated that the prognosis following radical surgery was superior to that following palliative surgery and no surgery. This finding is in line with a study conducted by Baratti, D et al. (32), which indicated that in cases that do not lead to severe postoperative complication rates and mortality rates, total parietal peritonectomy is feasible to maximize control of local lesions (33).The results (34) of a multicenter Phase III clinical study involving 456 patients with pleural mesothelioma established the combination of cisplatin and pemetrexed as the initial standard chemotherapy regimen for mesothelioma. Our study also indicates that chemotherapy is an effective treatment for extending overall survival in patients with peritoneal malignant mesothelioma. The treatment of malignant peritoneal mesothelioma was significantly associated with the stage. For the first time, we incorporated organ metastases into our survival prediction model for MPM, indicating that the prognosis for two or more organ metastases is much poorer than for single organ metastases. Overall, this nomogram can assist physicians and patients in predicting the survival of malignant peritoneal mesothelioma, enabling more personalized clinical management decisions to be made.

During nomogram evaluation, the C-index, calibration curve, and K-M curve demonstrated satisfactory discriminability and accuracy across 3 cohorts. Based on the demarcation point of the training cohort, patients were stratified into high and low risk groups. According to the cut-off point of patients in the training cohort, each group is divided into high and low-risk groups, which can clarify the overall survival stratification and provide auxiliary information for subsequent treatment. The clinician collects the key information of the patient and brings it into the nomogram to obtain the corresponding prognosis score. According to the score, the disease trend is judged, and the personalized treatment is carried out in combination with the guide to realize the organic combination of the nomogram and the treatment guide. High-risk patients should be watched more closely during the follow-up visit.

However, the study is subject to certain limitations. Firstly, within the SEER database, it is challenging to differentiate between “no” and “unknown” processing values. Given that our study involves retrospective data collection, it appears inevitable that there will be missing values in the data acquisition process, which will inevitably impact the analysis results. The following are some common methods to improve the integrity of the manuscript and supplement the missing values: ‘deletion method. If the proportion of missing values is relatively small, the case of deletion of missing values can be considered; the interpolation method uses different interpolation methods for different variables to make the model more in line with the distribution of the original data. Model prediction method, using the prediction model to estimate the missing value; the K-nearest neighbor algorithm estimates the corresponding variable values of K samples according to the ‘ distance ‘ UI in the sample space where the missing value is located. By changing the filling method of missing values or assuming different missing data, the changes of prognostic indicators such as survival probability of patients predicted by nomogram were observed. ‘Furthermore, additional information regarding diagnosis and treatment processes should be taken into consideration when selecting nomogram predictors, such as genetic tests (35), immunotherapy (36), molecular-targeted therapy (37), etc. Unfortunately, no data on these variables has been collected in the SEER database thus far. Additionally, this study is a retrospective study based on the SEER database and the Chinese cohort; the model’s applicability to the world is still being determined due to the differences in healthcare systems, disease epidemiology, early screening, and treatment patterns in different countries. Lastly, from a broader perspective we anticipate validating nomograms in prospective cohorts to enhance their stability and utility. Although the SEER database provides rich data for development, the heterogeneity of the external validation cohort (such as racial differences and marital status differences) may affect the accuracy of the model, so the applicability of the prognostic model in different populations needs to be interpreted cautiously.

## 5 Conclusion

In summary of this study’s findings indicate that age, sex, histology, surgery, tumor size, chemotherapy, differentiation and number of organ metastases were identified as independent factors for overall survival (OS) among MPM patients. Based on these factors we have developed a nomogram which can be utilized to assess prognosis for malignant peritoneal mesothelioma patients. External validation has confirmed its strong performance and practicality across different ethnic groups providing valuable guidance for clinical decision-making and enabling personalized care for patients with malignant mesothelioma.

## Data Availability

The original contributions presented in the study are included in the article/**Supplementary Material**. Further inquiries can be directed to the corresponding author.
