# Supplementary material for: Development and validation of a prognostic nomogram for patients with malignant peritoneal mesothelioma
Source: Front Oncol. 2025 Feb 28;15:1480197. doi: 10.3389/fonc.2025.1480197 (PMC11906319; doi:10.3389/fonc.2025.1480197)
Supplement: Supplementary file 1 [file DataSheet1.docx]

**Figure S1.** X-tile software determines optimal cutoff for age. (a) age distribution histogram and optimal cutoff value; (b) survival curves based on optimal cutoff values.


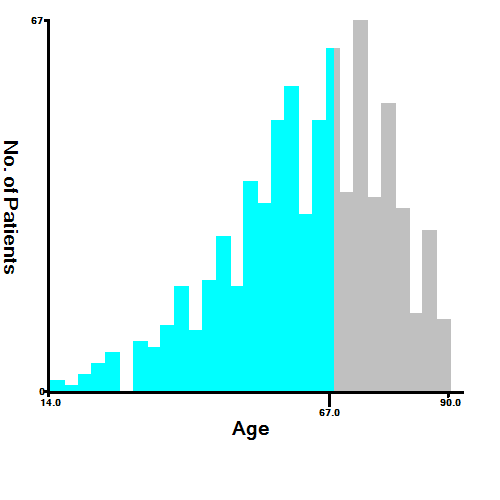

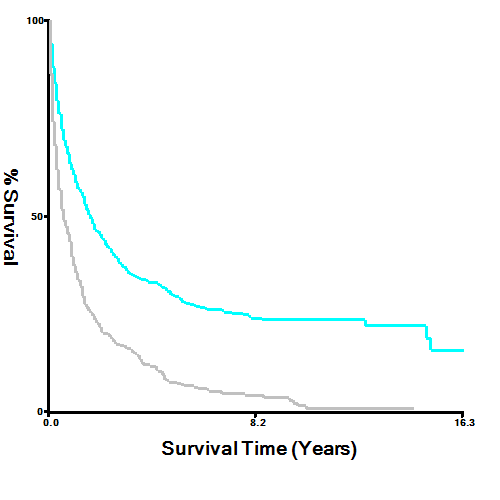


(a) (b)

**Figure S2.** X-tile software determines optimal cutoff for tumor size. (a) tumor size distribution histogram and optimal cutoff value; (b) survival curves based on optimal cutoff values.


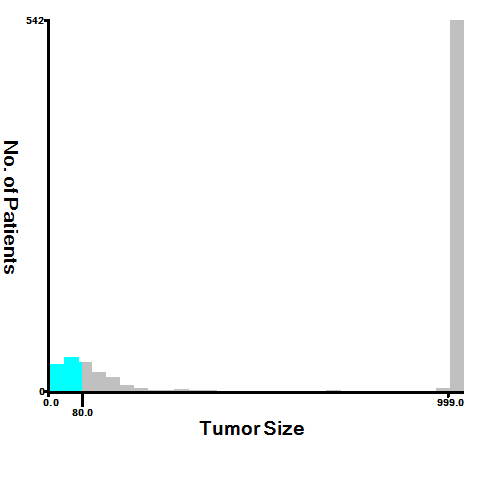

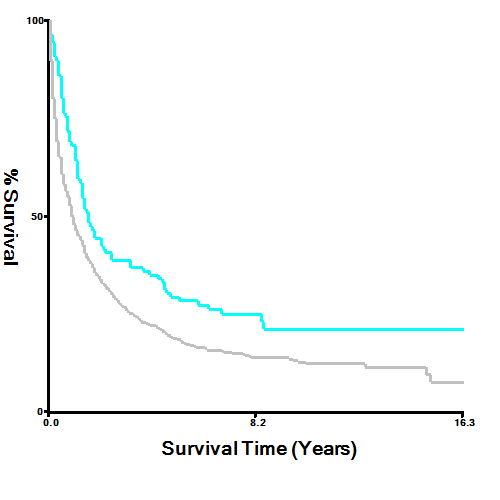


(a) (b)

**Figure S3.** X-tile software determines optimal cutoff for the total score . (a)the total score distribution histogram and optimal cutoff value; (b) survival curves based on optimal cutoff values.


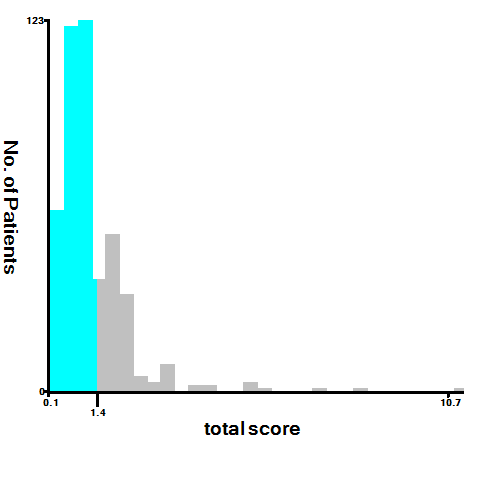

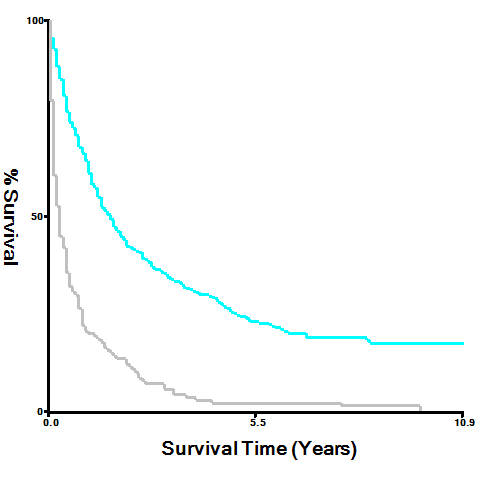


(a) (b)

**Table S1.** Univariate and multivariate Cox regression analysis of overall survival in the training cohort

| Dependent | | all | HR (univariable) | HR (multivariable) | HR (final) |
| --- | --- | --- | --- | --- | --- |
| age | ≥67 | 76 (16.8%) |  |  |  |
|  | 18-67 | 377 (83.2%) | 0.49 (0.38-0.63, p<.001) | 0.76 (0.57-1.01, p=.058) | 0.76 (0.58-1.01, p=.057) |
| sex | Female | 206 (45.5%) |  |  |  |
|  | Male | 247 (54.5%) | 1.52 (1.24-1.86, p<.001) | 1.46 (1.18-1.81, p<.001) | 1.43 (1.16-1.77, p=.001) |
| race | Black | 25 (5.5%) |  |  |  |
|  | Other | 30 (6.6%) | 0.83 (0.46-1.48, p=.527) |  |  |
|  | White | 398 (87.9%) | 0.89 (0.57-1.38, p=.599) |  |  |
| histology | Biphasic | 18 (4.0%) |  |  |  |
|  | Epithelial | 193 (42.6%) | 0.59 (0.35-0.99, p=.045) | 0.54 (0.32-0.92, p=.024) | 0.54 (0.32-0.92, p=.024) |
|  | Fibrous | 19 (4.2%) | 1.16 (0.59-2.30, p=.669) | 0.58 (0.28-1.18, p=.132) |  |
|  | NOS | 223 (49.2%) | 0.73 (0.44-1.22, p=.236) | 0.56 (0.33-0.96, p=.035) | 0.56 (0.33-0.96, p=.035) |
| primary.site | Overlapping lesion of retroperitoneum & peritoneum | 2 (0.4%) |  |  |  |
|  | Peritoneum | 440 (97.1%) | 0.69 (0.17-2.79, p=.607) |  |  |
|  | Retroperitoneum | 11 (2.4%) | 0.55 (0.12-2.56, p=.448) |  |  |
| surgery | None | 283 (62.5%) |  |  |  |
|  | NOS | 2 (0.4%) | 0.20 (0.03-1.41, p=.106) | 0.34 (0.05-2.47, p=.288) | 0.34 (0.05-2.44, p=.281) |
|  | Palliative | 133 (29.4%) | 0.44 (0.35-0.56, p<.001) | 0.52 (0.40-0.67, p<.001) | 0.52 (0.41-0.67, p<.001) |
|  | Radical | 35 (7.7%) | 0.56 (0.38-0.82, p=.003) | 0.62 (0.42-0.93, p=.022) | 0.65 (0.44-0.96, p=.031) |
| laterality | Bilateral | 9 (2.0%) |  |  |  |
|  | Unilateral | 444 (98.0%) | 0.90 (0.43-1.91, p=.791) |  |  |
| tumor.Size | ＜80 | 23 (5.1%) |  |  |  |
|  | ≥80 | 430 (94.9%) | 1.90 (1.13-3.19, p=.015) | 2.35 (1.35-4.09, p=.002) | 2.37 (1.36-4.12, p=.002) |
| marital.status | Divorced | 41 (9.1%) |  |  |  |
|  | Married | 260 (57.4%) | 0.91 (0.65-1.28, p=.602) |  |  |
|  | Other | 152 (33.6%) | 0.90 (0.63-1.28, p=.546) |  |  |
| radiation | No/Unknown | 449 (99.1%) |  |  |  |
|  | Yes | 4 (0.9%) | 1.78 (0.66-4.80, p=.251) |  |  |
| chemotherapy | No/Unknown | 173 (38.2%) |  |  |  |
|  | Yes | 280 (61.8%) | 0.77 (0.63-0.94, p=.012) | 0.67 (0.54-0.84, p=.001) | 0.67 (0.53-0.83, p<.001) |
| differentiated | Moderately | 4 (0.9%) |  |  |  |
|  | Poorly | 26 (5.7%) | 1.84 (0.64-5.29, p=.259) | 1.82 (0.62-5.32, p=.274) | 2.02 (0.70-5.85, p=.196) |
|  | Undifferentiated | 7 (1.5%) | 1.21 (0.34-4.30, p=.766) | 1.43 (0.39-5.26, p=.590) | 1.50 (0.41-5.49, p=.543) |
|  | Unknown | 392 (86.5%) | 1.07 (0.40-2.87, p=.890) | 1.05 (0.39-2.84, p=.926) | 1.06 (0.39-2.87, p=.904) |
|  | Well | 24 (5.3%) | 0.24 (0.08-0.77, p=.016) | 0.27 (0.08-0.87, p=.028) | 0.26 (0.08-0.86, p=.026) |
| t.stage | T1 | 1 (0.2%) |  |  |  |
|  | T2 | 12 (2.6%) | 2.27(0.29-17.50, p=.431) |  |  |
|  | T3 | 38 (8.4%) | 2.26(0.31-16.52, p=.420) |  |  |
|  | TX | 402 (88.7%) | 1.04 (0.15-7.39, p=.972) |  |  |
| n.stage | N0 | 68 (15.0%) |  |  |  |
|  | N1 | 8 (1.8%) | 1.19 (0.57-2.47, p=.646) | 1.20 (0.57-2.55, p=.629) |  |
|  | NX | 377 (83.2%) | 0.54 (0.41-0.71, p<.001) | 0.87 (0.46-1.65, p=.664) |  |
| m.stage | M0 | 37 (8.2%) |  |  |  |
|  | M1 | 51 (11.3%) | 1.06 (0.70-1.63, p=.773) | 1.00 (0.63-1.59, p=.986) |  |
|  | MX | 365 (80.6%) | 0.57 (0.41-0.81, p=.002) | 0.78 (0.42-1.45, p=.436) |  |
| bone.metastasis | No | 449 (99.1%) |  |  |  |
|  | Yes | 4 (0.9%) | 2.59 (0.96-6.94, p=.059) |  |  |
| liver.metastasis | No | 410 (90.5%) |  |  |  |
|  | Yes | 43 (9.5%) | 1.33 (0.95-1.84, p=.092) |  |  |
| lung.metastasis | No | 435 (96.0%) |  |  |  |
|  | Yes | 18 (4.0%) | 1.66 (1.01-2.75, p=.047) | 1.03 (0.54-1.95, p=.924) |  |
| number.of.organ.metastases | one | 58 (12.8%) |  |  |  |
|  | two | 6 (1.3%) | 4.52 (1.92-10.64, p<.001) | 4.07 (1.58-10.45, p=.004) | 4.17(1.74-10.02, p=.001) |
|  | zero | 389 (85.9%) | 0.84 (0.63-1.13, p=.258) | 0.86 (0.61-1.22, p=.406) | 0.86 (0.63-1.18, p=.364) |
